# Supplementary material for: Thermal Pyrolysis of Polystyrene Aided by a Nitroxide End-Functionality Improved Process and Modeling of the Full Molecular Weight Distribution
Source: Polymers (Basel). 2021 Dec 31;14(1):160. doi: 10.3390/polym14010160 (PMC8747164; doi:10.3390/polym14010160)
Supplement: Supplementary file 1 [file polymers-14-00160-s001.zip › polymers-1525419-supplementary.pdf]

Supplementary Material for

Thermal Pyrolysis of Polystyrene Aided by a Nitroxide End-functionality. Improved Process and Modeling of the Full Molecular Weight Distribution

Antonio Monroy-Alonso[1], Almendra Ordaz-Quintero[1], Jorge C. Ramirez[1], E. Saldívar-Guerra[1]*

[1]Centro de Investigación en Química Aplicada, Polymerization Processes Dept. Blvd. Enrique Reyna 140, Saltillo Coahuila, 25192, México

Polystyrene Polymerization

Polystyrene samples were synthesized in two different ways, by the Nitroxide Mediated Polymerization (NMP) and the Free Radical Polymerization (FRP) techniques, each one used different reaction temperatures and took contrasting polymerization times; yet, both aimed to obtain a molecular weight in number (M_n) of 50,000 Da. All polymerizations were conducted in bulk using a batch process. The process and conditions were identical to those described in a previous publication of our group [1]. Polymer samples were prepared with different nitroxide to initiator ratios to indirectly evaluate the effect of the nitroxide functionality level on the pyrolysis reaction (N/I= 0, 0.9, 1.1, 1.3). As in our previous work, the polymer samples obtained by polymerization were dissolved in acetone and precipitated in methanol to remove residual monomer, filtered, and air-dried obtaining a fine powder as a final product. For each performed polymerization, conversion was calculated by gravimetry at the end of the reaction. The synthesized polymer samples were analyzed using a gel permeation chromatograph (PL-GPC 50 Agilent Technologies) equipped with a PLGEL 5 µm mixed C column coupled with a refraction index detector to determine their MWD.

Polystyrene Pyrolysis (detailed description)

The used depolymerization reaction system, shown in Figure 1, consists of a 50 ml stainless steel reactor vessel that is heated homogeneously by an electric mantle. The temperature is accurately measured and controlled with the help of a thermocouple located inside the reactor and an on-off temperature controller that governs the flow of electrical current to the heating mantle through a 900 W rheostat set to work at its maximum voltage (200 V). The reactor is fitted with a manometer and two distinct valves; one connected to a nitrogen source and the other, to a condenser. To ensure that the condenser temperature is maintained at -3 °C during the experiments, allowing the condensation of some of the pyrolysis products with exception of non-condensable gases, a cooling bath that provides a glycol-water cooling mix, was also used. The system is almost identical to the one used in our previous studies [1], with the important difference that all the valves and connections of the reactor vessel are welded (or perfectly Teflon^{TM}-sealed), making a complete unit and preventing the produced reaction fumes from escaping the system from other places than from the condenser.

The pyrolysis experiments were performed in two stages, first, a set of exploratory pyrolysis reactions were conducted to evaluate the performance of the pyrolysis system that led to defining a set of reaction conditions and a process that involves fixed temperature ramps that help to maximize the amount of monomer recovery on the product liquid fraction. Then, using the previously established temperature ramps and set of conditions, pyrolysis reactions were performed using the synthesized polystyrenes.

The procedure for the pyrolysis of PS that was refined from the exploratory experiments is very similar to that reported before by us with one important difference. The process can be summarized as follows: 10 grams of polymer powder were placed in the reaction vessel and

nitrogen gas was passed through the system to remove oxygen from inside the reactor and maintain a final light positive pressure of 0.1 psig, then all system valves were closed and sequential well-defined temperature ramps were applied through the heating mantle to reach the desired pyrolysis temperatures. The current provided to the resistance in the heating mantle was regulated by a 900 Watt rheostat. The key important difference with respect to the process previously reported by us lies in the heating ramps used in this case in which the heating procedure was implemented as follows:

The temperature controller was set in manual mode and its set-point was initially set at 50 °C. When the temperature of the reactor reached 35 °C (from the initial value of ambient temperature 18-22 °C) the set-point was set at 100 °C and when 85 °C was reached a third set-point at 150 °C was programmed. Given the thermal inertia of the system, the temperature surpassed the 150 °C of the set-point and reached a value in the range 300-330 °C without further heating. The temperature control system consisted only of a heating mantle, so when the set-point was reached, the heating stopped, but there was not a cooling device to avoid further increase of the temperature. Once the temperature of the system stabilized around 300-330 °C, the set-point was set at 345 °C and the heat provided in this stage was sufficient to reach 390 °C for pyrolysis reactions designed to be run at this temperature. If the designed reaction temperature was 420 °C, once the system reached 390 °C the set-point was changed to ~400 °C and this was sufficient to reach the target temperature (see Table 1 for a summary of the procedure). In all the cases the rheostat was set to work at its maximum voltage (220 V) so it is possible to calculate the supplied power for scaling-up purposes. During most of the heating stage, the valve between the reactor and the condenser remained closed. Only

when the pressure of the system reached 12 psig and the temperature was between 330-350 °C was the valve open to allow the flow of vapors to the condenser.

If the system temperature is allowed to decrease, recombination of the aromatic components is induced and no liquid recovery is achieved (liquids are recovered at 385°C). Once the final reaction temperature is reached, it is maintained constant by setting the (on-off) controller in automatic mode at the desired set-point until all the material pyrolyzes; this allows to control the temperature within 5 °C around the target. The reaction was considered finished once no more liquid material was recovered from the condenser. It is important to point out that if heating ramps with ΔT higher than 15°C are used from 320°C on, recombination of light components will predominate, diminishing the amount of monomer present in the liquid phase. Even though solid, liquid, and gas products are produced during the pyrolysis reaction, only the liquid phase was recovered at the end of the reaction.

Each synthesized polystyrene sample (FRP and NMP) was submitted to the previously described process at two different maximum pyrolysis temperatures: 390°C and 420°C, (the previous study also used 450°C). Solids, liquids, and gases were obtained as products, but only the liquid fraction was recovered and characterized while the solids were only weighed.

The liquid phase obtained from the pyrolysis was analyzed and characterized by gas chromatography-mass spectrometry in an Agilent Technologies equipment, model 7890 GC/MSD 5977B fitted with an HP-5MS-30m column of 0.25 mm internal diameter, 0.25 µm of pore size (Agilent Technologies), and calibrated with a PFTBA standard. The sample injection volume was 1.5 mL with helium as the carrier gas with a flux of 1 mL/minute. The injection temperature was 250 °C and the sample running temperature was from 80 to 300°C (10°C/min).

Kinetic Mechanism

Mid Chain random scission, dormant polymer	$S_n \xrightarrow{k_b} P_n + R_{n-r}$
Mid chain random scission, dead polymer	$D_n \xrightarrow{k_b} P_r + P_{n-r}$
End chain scission, dead polymer	$D_n \xrightarrow{k_{be}} M\cdot + P_{n-1}$
End chain scission or activation, dormant polymer	$S_n \underset{k_d}{\overset{k_a}{\leftrightarrow}} N + P_n$
Transfer to polymer + β-scission (1)	$P_n + S_m \xrightarrow{k_{tr\beta}} D_n$<br>$\xrightarrow{½} P_{m-r} + S_r$<br>$\xrightarrow{½} R_{m-r} + D_r$
Transfer to polymer + β-scission (2)	$R_n + S_m \xrightarrow{k_{tr\beta}} S_n$<br>$\xrightarrow{½} P_{m-r} + S_r$<br>$\xrightarrow{½} R_{m-r} + D_r$
Transfer to polymer + β-scission (3)	$P_n + D_m \xrightarrow{k_{tr\beta}} D_n + P_{m-r} + D_r$
Transfer to polymer + β-scission (4)	$R_n + D_m \xrightarrow{k_{tr\beta}} S_n + P_{m-r} + D_r$
De-propagation	$P_m \xrightarrow{k_{rev}} P_{m-1} + M$<br>$R_m \xrightarrow{k_{rev}} R_{m-1} + M$
Termination by combination	$P_n + P_m \xrightarrow{k_{tc}} D_{n+m}$<br>$P_n + R_m \xrightarrow{k_{tc}} S_{n+m}$<br>$R_n + R_m \xrightarrow{k_{tc}} S_{n+m}$
Termination by disproportionation	$P_n + P_m \xrightarrow{k_{td}} D_n + D_m$<br>$P_n + R_m \xrightarrow{k_{td}} D_n + S_m$<br>$R_n + R_m \xrightarrow{k_{td}} S_n + S_m$
Termination with monomeric radicals	$P_n + M\cdot \xrightarrow{k_{tc}} D_{m+1}$<br>$P_n + M\cdot \xrightarrow{k_{td}}$ [illegible]

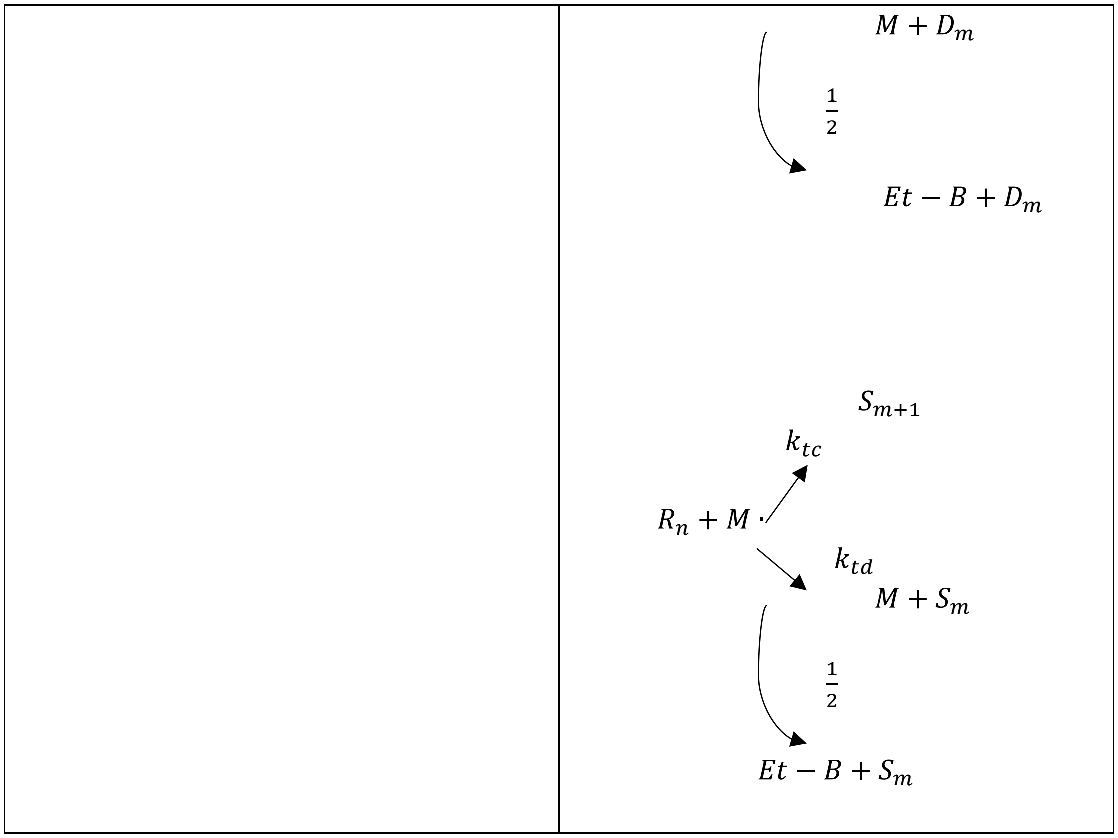

Properties of Synthesized Polystyrene

Table S1. *Properties of synthesized polystyrenes via FRP and NMP.*

**Sample**	**M_n (g/mol)**	**M_w (g/mol)**	**Dispersity (D)**	**Reaction time (minutes)**	**Conversion (%)**
**FRP**	48100	84300	1.75	140	82.8
**NMP, N/I =0.9**	37000	49300	1.34	315	97.1
**NMP, N/I =1.1**	40000	52100	1.30	435	97.5
**NMP, N/I =1.3**	43300	54300	1.25	510	97.4

Polymer Molecular Weight Distributions and Function Fitting for Initial Conditions of Simulations

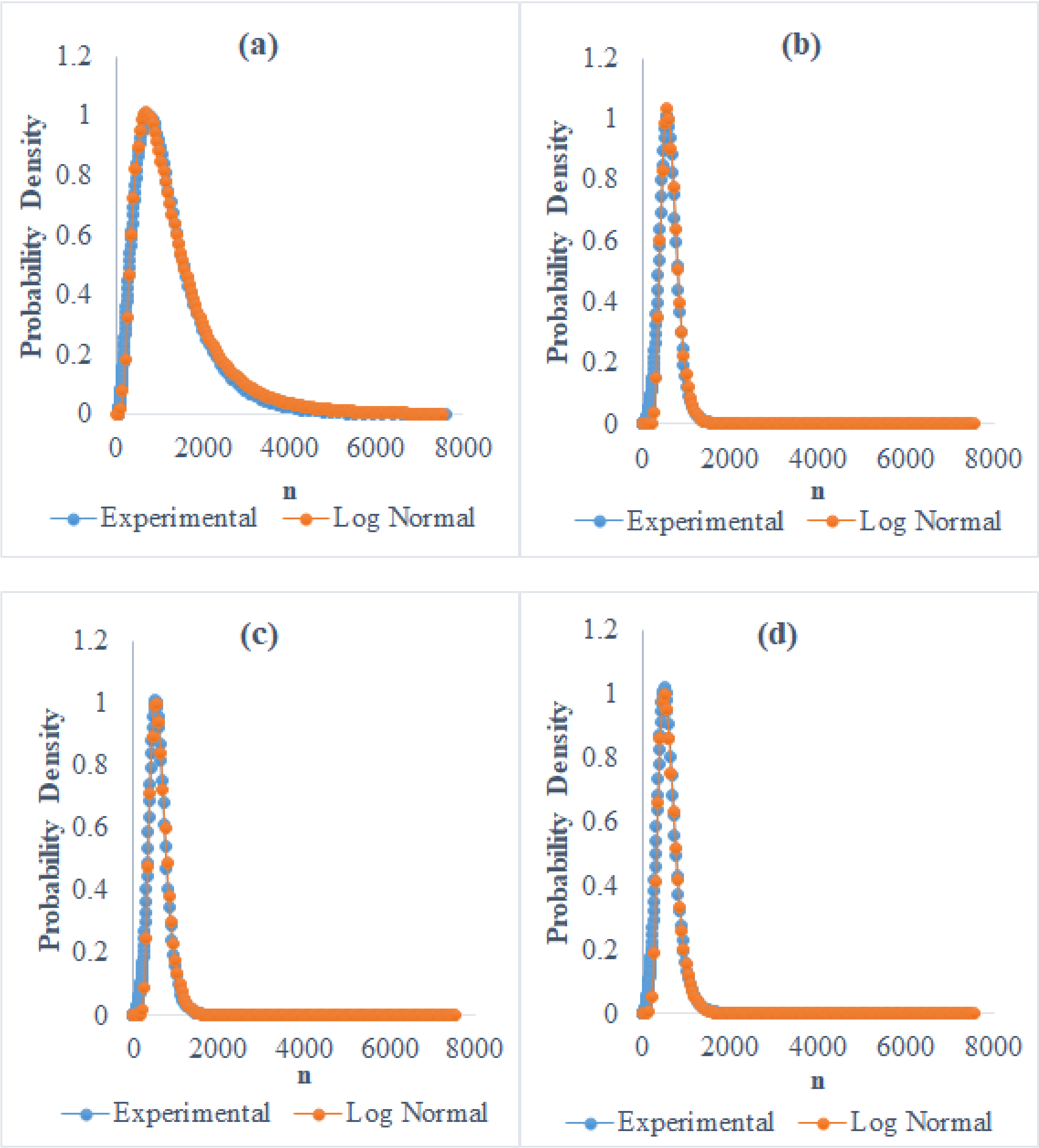

Figure S1. *Molecular Weight Distribution of synthesized polystyrenes by a) FRP, b) NMP, N/I=1.3, c) NMP, N/I=1.1, d) NMP, N/I=0.9, used as initial conditions for the pyrolysis simulations.*
